# Comprehensive Analysis of the SBP Family in Blueberry and Their Regulatory Mechanism Controlling Chlorophyll Accumulation

**DOI:** 10.3389/fpls.2021.703994

**Published:** 2021-07-01

**Authors:** Xin Xie, Shaokang Yue, Baosheng Shi, Hongxue Li, Yuhai Cui, Jingying Wang, Pengjie Yang, Shuchun Li, Xuyan Li, Shaomin Bian

**Affiliations:** ^1^College of Plant Science, Jilin University, Changchun, China; ^2^College of Landscape Architecture and Tourism, Hebei Agricultural University, Baoding, China; ^3^London Research and Development Centre, Agriculture and Agri-Food Canada, London, ON Canada; ^4^Department of Biology, Western University, London, ON, Canada; ^5^Department of Pain, Second Hospital of Jilin University, Changchun, China

**Keywords:** blueberry, SBP gene, miR156, chlorophyll accumulation, SBP targets

## Abstract

SQUAMOSA Promoter Binding Protein (SBP) family genes act as central players to regulate plant growth and development with functional redundancy and specificity. Addressing the diversity of the SBP family in crops is of great significance to precisely utilize them to improve agronomic traits. Blueberry is an important economic berry crop. However, the SBP family has not been described in blueberry. In the present study, twenty *VcSBP* genes were identified through data mining against blueberry transcriptome databases. These VcSBPs could be clustered into eight groups, and the gene structures and motif compositions are divergent among the groups and similar within each group. The VcSBPs were differentially expressed in various tissues. Intriguingly, 10 VcSBPs were highly expressed at green fruit stages and dramatically decreased at the onset of fruit ripening, implying that they are important regulators during early fruit development. Computational analysis showed that 10 *VcSBP*s were targeted by miR156, and four of them were further verified by degradome sequencing. Moreover, their functional diversity was studied in Arabidopsis. Noticeably, three VcSBPs significantly increased chlorophyll accumulation, and qRT-PCR analysis indicated that VcSBP13a in Arabidopsis enhanced the expression of chlorophyll biosynthetic genes such as AtDVR, AtPORA, AtPORB, AtPORC, and AtCAO. Finally, the targets of VcSBPs were computationally identified in blueberry, and the Y1H assay showed that VcSBP13a could physically bind to the promoter region of the chlorophyll-associated gene *VcLHCB1*. Our findings provided an overall framework for individually understanding the characteristics and functions of the SBP family in blueberry.

## Introduction

Blueberry (*Vaccinium* spp.) is a globally cultivated perennial shrub with outstanding economic value. Its fruit is not only sweet but also rich in nutrients, especially anthocyanins, which greatly promote human health such as improvement of vision, blood glucose balance, elimination of free radicals, aging delay, inhibition of obesity and hyperlipidemia, and prevention of cardiovascular diseases (Routray and Orsat, 2011). Thus, blueberry growth and development, especially the events related to fruit ripening and anthocyanin biosynthesis, have started to attract attention in recent years. To date, a few regulators have been shown to be involved in the regulation of blueberry growth and development, including transcription factor genes *VcMYB*s, *VcSOC1-k*, *VcDDF1*, *VcFT*, some miRNAs, and hormones (IAA and ABA) (Zifkin et al., 2012; Song et al., 2013; Walworth et al., 2016; Hou et al., 2017, 2020; Song and Gao, 2017; Plunkett et al., 2018). Recently, high-throughput sequencing data provided considerable information for identifying and characterizing the regulators that control blueberry growth and development (Rowland et al., 2012; Hou et al., 2017; Qi et al., 2019). However, our understanding of the regulatory network underlying blueberry growth and development are extremely limited.

SQUAMOSA Promoter Binding Proteins (SBPs) constitute a plant-specific transcription factor family featured by a highly conserved SBP domain of 76 amino acids. Generally, the SBP domain harbors three common structures: two tandem zinc fingers (C3H and C2HC) and a nuclear localization signal (NLS), partially overlapping with the second zinc finger at the C-terminal (Birkenbihl et al., 2005). It has been well known that SBP proteins can bind to a consensus DNA sequence TNCGTACAA with the GTAC as the binding core, therefore regulating the expression of their target clients (Birkenbihl et al., 2005; Kropat et al., 2005). SBP proteins play important roles in various biological and cellular processes through regulating their target clients, spanning virtually every aspect of plant growth and development as well as stress response. These include leaf morphology and leaf initiation (Preston et al., 2016), trichome formation (Yu et al., 2010), phase transition (Xu et al., 2016), shoot branching and maturation (Gao et al., 2018), regeneration of shoot and root (Barrera-Rojas et al., 2020; Ye et al., 2020), root development (Yu et al., 2015), flowering (Xie et al., 2020), male fertility (Xing et al., 2010), ovary and fruit development (Silva et al., 2014), cell number and size (Usami et al., 2009), and grain yield (Wang et al., 2017), etc. Evidently, *SBP* genes are a class of central players in the regulation of plant growth and development, which can be utilized for the improvement of important agronomic traits.

In 1996, the first two *SBP*s were identified in *Antirrhinum majus*, and shown to regulate the expression of the MADS-box gene *SQUAMOSA* directly through binding to its promoter region, therefore controlling flowering (Klein et al., 1996). With the availability of whole-genome information and transcriptome data, *SBP* genes have been isolated in many plant species, from the model plant Arabidopsis to economically important crops (Salinas et al., 2012; Hou et al., 2013; Bhogale et al., 2014; Li and Lu, 2014; Shalom et al., 2015). The SBP family is a relatively small group of transcription factors in plants, and the SBP family members show diverse features and evolutionary divergences. Emerging evidence indicated that SBPs exert their regulatory functions in a member-specific manner. For example, *SBP-like 9* (*SPL9*) in *Arabidopsis thaliana* might serve as a negative regulator of wall ingrowth deposition in transfer cells of phloem parenchyma (Nguyen et al., 2017), whereas *SPL3* cannot affect the deposition of wall ingrowth but enhance phosphate-deficient response (Lei et al., 2016). Likewise, *OsSPL14* acts in controlling rice tillering growth (Luo et al., 2012), and *OsSPL16* was found to be a regulator of grain size, shape, and quality in *Oryza sativa* (Wang et al., 2012). Nevertheless, a number of studies showed that members of the SBP family could be functionally redundant in the regulation of plant growth and development. For example, *AtSPL3/4/5* redundantly promote flowering through activating the expression of *LEAFY*, *FRUITFULL*, and *APETALA1* (Jung et al., 2016), while *AtSPL9/15* and *AtSPL2/10/11* act as regulators of plastochron and branching (Schwarz et al., 2008; Shikata et al., 2009). Additionally, a subset of *SBP* genes can be subjected to miR156-guided transcriptional cleavage and translational repression, for example, 11 out of the 17 *SPL*s in Arabidopsis and seven out of the 19 *SPL*s in pear, thereby being integrated into miR156/*SPL* modules to regulate plant growth, development, and stress response (Zhang et al., 2015; Qian et al., 2017). Clearly, the SBP family members show distinct features and perform their functions with redundancy and specificity. Thus, addressing the diversity and specificity of the SBP family in different crop species is of great significance in order to precisely utilize them to improve agronomic traits.

It has been accepted that the functional roles of *SBP* genes are highly conserved across plant species. However, novel functions of *SBP* genes have been constantly revealed in crop species with special developmental processes or organs (Bhogale et al., 2014; Silva et al., 2014; Qian et al., 2017). Fruit growth and ripening is a specific process for fruit-bearing plant species, and many fruit-specific events occur during the process. Accumulating evidence indicates that *SBP* genes are involved in the regulation of fruit growth and ripening. For instance, the Colorless non-ripening (CNR) locus of tomato (a homolog of *AtSPL3*) is crucial for fruit ripening (Manning et al., 2006), while *SPL18* in grape might regulate berry development at the veraison stage in an ABA-independent manner (Xie et al., 2019). Likewise, *VmTDR4* (a *SQUAMOSA*-class *MADS-box* gene) is positively involved in the regulation of anthocyanin accumulation during bilberry fruit ripening (Jaakola et al., 2010), while MaSPL16 in banana regulates carotenoid biosynthesis through promoting the expression of *MaLCYB*s genes (Zhu et al., 2020). These functions were not observed in non-fleshy-fruited plant species such as Arabidopsis and rice. Obviously, it is of great interest to comprehensively characterize the SBP family in crops with special developmental processes or organs and to reveal their functional roles and neo-functionalization.

Since the SBP family proteins are powerful regulators with functional diversification in plants, study of these genes will enhance understanding of the regulatory network underlying blueberry growth and development. To date, however, the characteristics and functional diversity of the SBP family have remained unexplored in blueberry. In recent years, the transcriptional profiles of blueberry leaves, flower buds, and fruits at different development stages have been investigated using high-throughput sequencing technology (Rowland et al., 2012; Gupta et al., 2015; Li et al., 2016). These transcriptome data have enabled the identification of the *SBP* family genes involved in blueberry growth and development. In the present study, 20 *VcSBP*s were identified from the blueberry transcriptome database. Gene structure, phylogeny, motif composition, miRNA target sites, and expression patterns in different tissues were systematically analyzed. Furthermore, the functional diversity of the *VcSBP* family genes were studied in Arabidopsis. Additionally, the targets of VcSBP proteins were investigated in blueberry. These findings lay a foundation for further studying the functional roles of the *SBP* genes and their regulatory mechanisms during blueberry growth and development, which will contribute to the improvement of blueberry agronomic traits.

## Materials and Methods

### Plant Materials

Seven-year-old blueberry trees (*Vaccinium corymbosum*, cv. Northland) from clonal propagation were grown at the experimental station at Jilin University (Changchun, China). Blueberry tissues were randomly harvested from six different seven-year-old blueberry plants, including new leaf, young shoot, unopened flower, opening flower, and fruit at six developmental stages [green pad (FS1), green cup I (FS2), green cup II (FS3), light green/white (FWS), pink (FPS) and blue (FMS) fruits] (Li et al., 2020), frozen in liquid nitrogen and stored at −80°C.

Arabidopsis and tobacco (*Nicotiana benthamiana*) plants were grown in growth chambers under long-days (16 h light/8 h dark) at 20°C with 70–80% relative humidity.

### Identification of *SBP* Genes in Blueberry

The CDS sequences of *SBP* genes from Arabidopsis and grape were downloaded from the publicly available databases TAIR^1^ or Phytozome^2^, and then used as reference sequences to perform local blast searches for querying their homologs against the publicly available transcriptome databases of blueberry^3^ and our previously assembled transcriptome data. The conserved SBP-specific domains were confirmed using the PROSITE Server^4^, and all of the *SBP-like* genes without an SBP domain were discarded. The physicochemical properties, including molecular weight (MW), and isoelectric point (pI), of the identified SBP proteins, were predicted using the ExPASy Compute pI/Mw tool^5^.

### Chromosomal Location and Phylogenetic Analysis of the *VcSBP* Family Genes

All *VcSBP* genes were mapped to the genome of *V. corymbosum*, cv. Draper, according to the approximate location information (Colle et al., 2019), and their positions were imported into the CIRCOS software to generate a circle plot (Krzywinski et al., 2009). The SBP protein sequences (17 from grape, 27 from apple, and 17 from tomato) were downloaded from Phytozome (see text footnote 2). All the SBP protein sequences from blueberry, Arabidopsis, grape, apple, and tomato were used for phylogenetic analysis, and phylogenetic trees were constructed with the MEGA7.0 software using the maximum likelihood with 1000 bootstrap replications (Kumar et al., 2016). The sequence logo was created using Weblogo online software^6^.

### Analysis of Gene Structure and Conserved Protein Motifs

The exon/intron structure of each *VcSBP* gene was analyzed using the Gene Structure Display Server^7^ by comparing the coding sequence and genomic sequence. Potentially conserved motifs of VcSBP proteins were predicted using the online Multiple Expectation Maximization for Motif Elucidation (MEME) toolkit^8^, with the following parameter settings: the minimum motif width = 20, the maximum motif width = 50, and the maximum number of motifs = 20.

### MicroRNA Target Prediction

To identify *VcSBP*s targeted by miR156/157, the coding regions and 3′ UTRs of all *VcSBP* sequences were analyzed at the psRNATarget server^9^ with blueberry miR156/157 mature sequences (Hou et al., 2017). The sequence logo of miR156/157 was created using the Weblogo online software (see text footnote 6).

### Expression *P*attern *A*nalysis of VcSBP *G*enes in *B*lueberry

Total RNAs were isolated from blueberry leaf, shoot, unopened flower, opening flower, fruit tissues at six developmental stages, and blueberry tissue culture seedlings as well as Arabidopsis leaf. First-strand cDNA was synthesized using the PrimeScript^TM^ RT reagent kit with gDNA Eraser (Takara, Japan). qRT-PCR was subsequently conducted with an ABI StepOnePlus PCR system and SYBR Premix Ex Taq (Takara, Japan). Blueberry *ACTIN* was set as an internal reference for data normalization. Three biological replicates with three technical replicates were performed for each sample, and data were analyzed by the software ABI StepOnePlus v2.3 and one-way ANOVA with LSD test, and *p*-value < 0.05 was considered to be statistically significant. Primer information is listed in Supplementary Table 1.

### Vector Construction and Plant Transformation

The full-length CDS of each *VcSBP* was amplified using gene-specific primers (Supplementary Table 1). All purified PCR products were cloned into the Gateway entry vector pDONR207 and then transferred into the destination vector pEarleyGate101 (pEG101) through homologous recombination. All the constructed plasmids were confirmed by PCR and sequencing. The expression vectors (pEG101*-VcSBP*s) were individually transformed into *Agrobacterium tumefaciens* strain GV3101.

Arabidopsis transformation was conducted using the floral dip method described by Zhang et al. (2006). Transgenic lines were screened in the soil with 200 μg/mL glufosinate and then confirmed by PCR with gene-specific primers. Primer information is listed in Supplementary Table 1.

The *VcMIR156a* gene was constructed into pBI121 as described previously (Li et al., 2020) and transferred into *Agrobacterium tumefaciens* strain EHA105. Blueberry transformation was performed according to the method described by Song and Sink (2006). The transgenic blueberry lines were obtained and confirmed by PCR with gene-specific primers (Supplementary Table 1). The *VcMIR156a*-overexpressing transgenic Arabidopsis were generated as previously described (Li et al., 2020).

### Prediction of *VcSBP* Targets in Blueberry

The genes containing the TNCGTACAA element within 2000 bp upstream were extracted against the reference genome of blueberry (*V. corymbosum*, cv. Draper) (Colle et al., 2019). To functionally annotate these targets, all protein sequences were analyzed using eggNOG-Mapper^10^. Density distribution of distance was visualized using ggplot2 in R.

### Yeast One-Hybrid (Y1H) Assay

To investigate the interaction of VcSBPs and their targets, the full-length CDS of *VcSBP13a* was cloned and introduced into the vector pB42AD. The fragment 597-796 bp upstream of the transcription start site (TSS) of *VcLHCB1* containing two TNCGTACAA elements and the fragment 1096–1548 upstream of the TSS of *VcLHCB2* with five GTAC elements were cloned as promoter regions (*pVcLHCB1* and *pVcLHCB2*) and constructed into the vector pLacZi, respectively. Three negative controls, i.e., *pB42AD/pLacZi*, *pB42AD-VcSBP13a/pLacZi*, and *pB42AD/pLacZi-pVcLHCB1*, *pB42AD/pLacZi-pVcLHCB2*, and one positive control, *pB42AD-AtRVE8/placZi-AtPRR5*, were also generated. Different plasmid combinations were separately co-transformed into the yeast cells (EGY48). The primers are listed in Supplementary Table 1.

## Results

### Identification of *SBP* Genes and Their Characterization in Blueberry

To identify *SBP* genes in blueberry, the CDS sequences of the *SBP* genes from both *Vitis vinifera* and Arabidopsis were used as queries to conduct BLASTn against the *V. corymbosum* GDV RefTrans V1 and our previously assembled transcriptome data (Hou et al., 2017). After removal of redundant sequences, a total of 22 *SBP* sequences were identified in blueberry, which are then named as *VcSBP* and each of them assigned a species number corresponding to their closest homolog in Arabidopsis (Supplementary Figure 1). To verify the sequences of the *VcSBP* genes, their full-length CDSs were amplified and sequenced, and the results showed that all the cloned *SBP* genes are indeed the same sequence as listed in the Genome Database for *V. corymbosum* cv. Draper v1.0.

**FIGURE 1:**
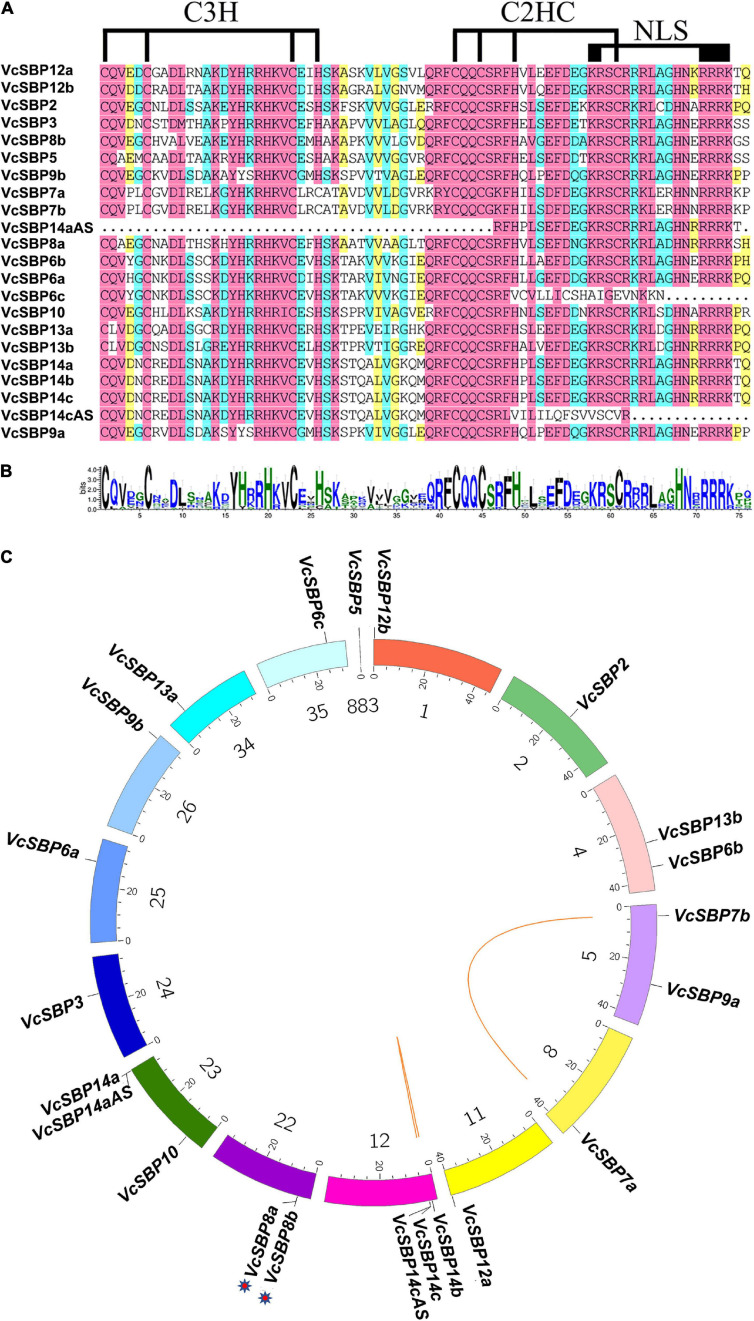
The SBP domains and chromosomal localization of the *VcSBP* family genes. **(A)** Multiple alignment of the SBP domains. The two conserved zinc-finger structures (C3H and C2HC) and the NLS are indicated. **(B)** Sequence logo of the SBP domains in VcSBPs. The total height of each stack represents the conservation degree of each position, while the height of the letters within each stack indicates the relative frequency of the corresponding amino acid. **(C)** Chromosomal localization and duplication of *SBP* genes in blueberry. Each colored box represents a scaffold. The approximate distribution of each *VcSBP* gene is marked on the circle with a short black line. The tandem duplication cluster is indicated with stars. Colored lines indicate the linkage group with segmental duplication.

The features of all the *VcSBP* family members were computationally characterized. As shown in Supplementary Table 2, the CDS lengths of these *VcSBP* genes are quite variable, ranging from 363 to 3222 bp, which is consistent with the *SBP* family in other plant species such as Arabidopsis, apple, grape (Hou et al., 2013; Li et al., 2013; Zhang et al., 2015). Their deduced proteins were estimated to possess the theoretical pI values from 5.96 to 10.54 and the MWs from 23.17 to 117.97 kDa. Furthermore, the SBP domains were analyzed using the online tool CD search^11^. As shown in Figures 1A,B, all the SBP proteins, except VcSBP6c, VcSBP14aAS, and VcSBP14cAS, contain a typical SBP domain featured by two zinc finger structures (C3H and C2HC) and an NLS motif. VcSBP14aAS harbors an SBP domain with the absence of C3H and an incomplete C2HC, while the SBP domains in VcSBP6c and VcSBP14cAS lack the NLS motif (Figures 1A,B). These results indicated that the 22 putative genes are SBP family members.

### Distribution of *VcSBP* Genes in the Blueberry Genome and Their Evolutionary Relationships

To date, the draft genome assembly of *V. corymbosum* contains 1760 scaffolds^12^. To map the locations of the *VcSBP* family genes in the draft genome, a Circos map was generated using the corresponding scaffolds where the *VcSBP* genes are situated. It turns out that they are unevenly distributed in 15 different scaffolds (i.e., 1, 2, 4, 5, 8, 11, 12, 22, 23, 24, 25, 26, 34, 35, 883, Figure 1C). Further observation indicated that two pairs of *VcSBP* genes (*VcSBP14a* and *VcSBP14aAS*, *VcSBP14c*, and *VcSBP14cAS*) were situated at the same loci with the similarity of 65.01 and 93.09%, respectively, indicating that they might be derived from different transcript splicing of the same genes. Thus, the 22 *SBP* sequences were likely derived from 20 *SBP* genes and two alternative splices. Gene family expansion can arise from gene duplication events such as tandem duplication and segmental duplication of chromosomal regions (Leister, 2004). Generally, tandem duplication refer to those closely related genes separated by the distance within 50 kb in the same chromosome. It was observed that the distance between *VcSBP8a* and *VcSBP8b* is 15,110 bp (Figure 1C and Supplementary Table 2), suggesting that they might be derived from tandem duplication. In contrast, the distances between *VcSBP7a* and *VcSBP7b* as well as *VcSBP14b* and *VcSBP14c* are relatively far from each other, and their similarities reach 99.27 and 94.26%, respectively, implying that they were possibly generated from segmental duplication.

To explore the evolutionary relationships among the SBP family proteins, a phylogenetic tree was generated using the protein sequences of VcSBPs and the SBPs from apple, grape, tomato, and Arabidopsis. As shown in Supplementary Figure 2, all the SBP proteins were classified into six different groups (G1-G6), and VcSBPs were separately distributed to the 6 groups, suggesting that the VcSBP family might have experienced evolutionary diversification similar to those in the other four plant species. For example, seven small VcSBP proteins with no more than 254 aa (VcSBP3, VcSBP5, VcSBP6c, VcSBP9a, VcSBP10, VcSBP14aAS, and VcSBP14cAS) were separately distributed into the six groups, while the large proteins with more than 800 aa (VcSBP14a, VcSBP12a, and VcSPB7a/7b) were clustered into G5 and G6, respectively. Further observation indicated that VcSBPs were closer to their homologs from apple, grape, Arabidopsis and/or tomato in the phylogenetic tree. For instance, VcSBP3 was grouped together with SlySBP3, AtSBP3, VvSBP9, CNR, and while VcSBP2 was distributed into the subgroup of SBP2/10/11 with the inclusion of VvSBP2, SlySBP2, and AtSBP2.

### *VcSBP* Family Shows Diverse Gene Structures and Motif Compositions

**FIGURE 2:**
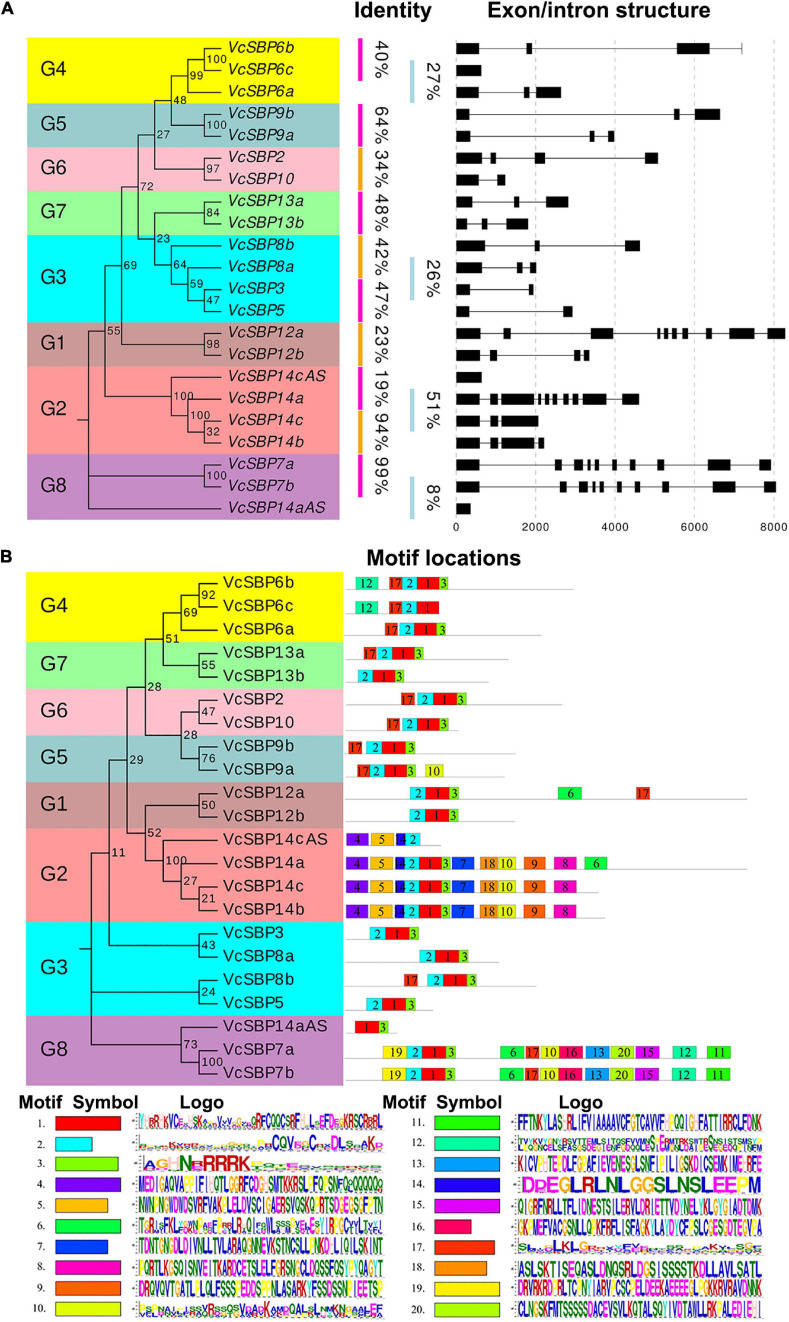
Gene structures and protein motif compositions of the VcSBP family. **(A)** The exon/intron structures of *VcSBP* genes. The left panel is the phylogenetic tree of *VcSBP* genes. Eight groups are clustered (G1–G8), and the percent similarity between the gene pair is listed. The right panel shows the intron-exon structures where the exons are shown by rectangular, and the introns are represented by thin lines. **(B)** Motif analysis of VcSBP proteins. The left panel is the phylogenetic tree of VcSBP proteins, and eight groups are clustered (G1–G8). The right panel shows the motif compositions of VcSBP proteins. The motifs were identified using the program MEME, represented with boxes of different colors labeled by number (1–20). The sequence logos of 20 motifs are listed, and the height of the letters within each stack indicates the relative frequency. The symbol and the ID number are corresponding to the colored box and the number in the right panel.

To understand the structural diversity of *VcSBP* family genes, the exon/intron structures were generated according to the gene coding and genomic sequences. Consistent with previous reports in other plant species (Hou et al., 2013; Li et al., 2013; Zhang et al., 2015), *VcSBP* genes showed a high variation in the number of exons. As indicated in Figure 2A, four *VcSBP* genes (*VcSBP7a*, *VcSBP7b*, *VcSBP12a*, and *VcSBP14a*) comprise 10 exons with intron intervals. In contrast, *VcSBP6c*, *VcSBP14aAS*, and *VcSBP14cAS* harbor only one exon without intron. The remaining *VcSBP*s have 2–4 exons. Furthermore, integration analysis of exon/intron structures with phylogenetic relationship and sequence identity was conducted. It turns out that the pairs of *VcSBP*s in the same clade basically display similar exon/intron structures (Figure 2A). Two pairs of duplicated genes (*VcSBP7a* and *VcSBP7b*, *VcSBP14b*, and *VcSBP14c*) show not only similar exon/intron structure but also high similarity with the values of 99.27 and 94.21%, respectively (Supplementary Table 2), supporting that they might undergo similar exon/intron gain or loss events with less functional diversification. However, the remaining *VcSBP* pairs with similar exon/intron structure in the same clade displayed relatively low similarities ranging from 8–48% (Figure 2A and Supplementary Table 2), implying the diversity of their functional roles.

To provide clues about the functional diversity of VcSBP family, conserved motifs in each of the VcSBP proteins were predicted using the online tool ScanProsite. As shown in Figure 2B, twenty conserved motifs were identified in VcSBPs, and two motifs (the motifs 1 and 2) constitute the SBP domain. Five VcSBPs (VcSBP7a/7b and VcSBP14a/14b/14c) harbor 11–13 motifs, while the remaining VcSBPs contain 2–5 motifs. Although most of the 20 motifs are functionally unknown, the existence of multiple motif compositions implied the functional diversity of the SBP family in blueberry. The 20 VcSBPs and two alternatively spliced species were clustered into eight groups in the phylogenetic tree. It was observed that the VcSBP proteins in the same group in the phylogenetic tree basically show similar motif composition, suggesting possible functional redundancy within the same group.

### *VcSBPs* Are Differentially Expressed in Different Tissues and Throughout Fruit Development

**FIGURE 3:**
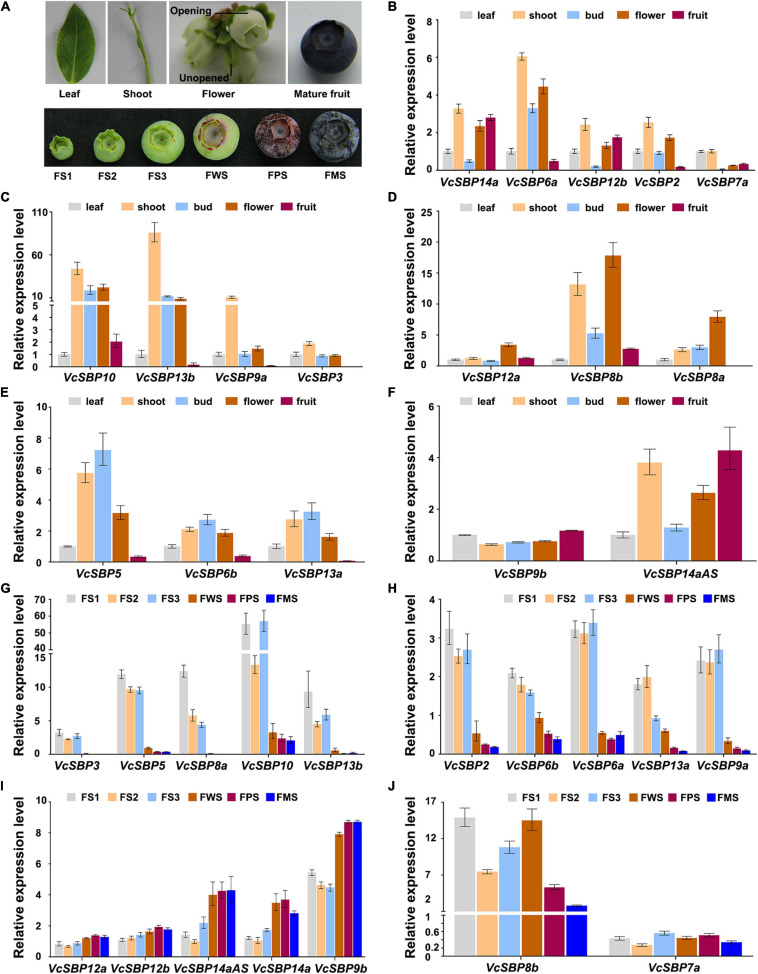
Expression patterns of *VcSBP* genes in different tissues and during fruit development. **(A)** Photograph of different tissues and fruits at six developmental stages. Different tissues include blueberry new leaf, young shoot, unopened flower, opening flower, and mature fruit, while FS1, FS2, FS3, FWS, FPS, and FMS refer to green pad, green cup I, green cup II, light green/white, pink, and blue stages, respectively. **(B–F)** Expression pattern of *VcSBP* genes in different blueberry tissues. **(G–J)** Expression pattern of *VcSBP* genes in fruits at six developmental stages. Total RNAs extracted from the above different tissues and fruits at six developmental stages were used for qRT-PCR analysis. Three biological replicates and three technical replicates for each biological replicate were performed. Error bars indicate standard errors. The data were normalized against the gene *VcACTIN*.

To obtain clues about the functional roles of *VcSBP* genes, their expression patterns in five tissues (new leaf, young shoot, opening, and unopened flower, and mature fruit; Figure 3A) were examined using qRT-PCR. Since high sequence similarity exists within each of the three *VcSBP* groups (*VcSBP7a/b*, *VcSBP6b/c*, and *VcSBP14a/b/c/cAS*), only one gene was chosen as representative for each group (*VcSBP6b*, *VcSBP7a*, and *VcSBP14a*). The examined *VcSBP* genes were found to be differentially expressed in the five tissues. As shown in Figures 3B,C, 10 *SBP* genes showed the highest expression in shoot, especially *VcSBP13b* and *VcSBP9a*, with 7.46–509.52 and 7.12–118.31-fold increase as compared to the other four tissues. Meanwhile, three *VcSBP* genes (*VcSBP8a*, *VcSBP8b*, and *VcSBP12a*) were highly expressed in opening flower (Figure 3D), and three *VcSBP* genes (*VcSBP5*, *VcSBP6b*, and *VcSBP13a*) in unopened flower and shoot (Figure 3E). In mature fruit, all the *VcSBP* genes were expressed at relatively low levels except *VcSBP9b*, *VcSBP12b*, *VcSBP14a*, and *VcSBP14aAS* (Figure 3F). These results suggested that the *VcSBP* family might perform functions in an organ-specific manner.

Blueberry fruit development can be generally divided into three phases: fruit growth, a transition from growth to maturation, and maturation (Zifkin et al., 2012). To explore the functional roles of *SBP* family during fruit development, the expression patterns of *VcSBP*s were investigated in fruits at six developmental stages (green pad, green cup I, green cup II, light green/white, pink, and blue fruit, Figure 3A). The three early developmental stages represent the growth phase; the light green/white stage corresponds to the transition stage; the pink and blue stages refer to the maturation phase. As shown in Figures 3G,H, 10 *VcSBP* genes were highly expressed at the three early developmental stages (green pad, green cup I, green cup II), and dramatically decreased at the light green stage (especially *VcSBP3*, *VcSBP5*, *VcSBP9b*, *VcSBP10*, and *VcSBP13b* with more than 10-fold changes as compared to the ones at green cup II), and then remained at a low level until fruit maturation. Conversely, the expression levels of some *VcSBP* genes (*VcSBP9b*, *VcSBP12a*, *VcSBP12b*, *VcSBP14a*, and *VcSBP14aAS*) were relatively low at the three early developmental stages, but then increased from the light green stage until fruit maturation (Figure 3I). Also, it was observed that *VcSBP8b* was gradually increased from the green cup I stage to the light green stage, and then remarkably decreased at the maturation stage (Figure 3J). These results suggested that *VcSBP* family might play different, even opposite, roles during blueberry fruit development.

### A Subset of *SBP* Genes Are Targeted by miR156 in Blueberry

**FIGURE 4:**
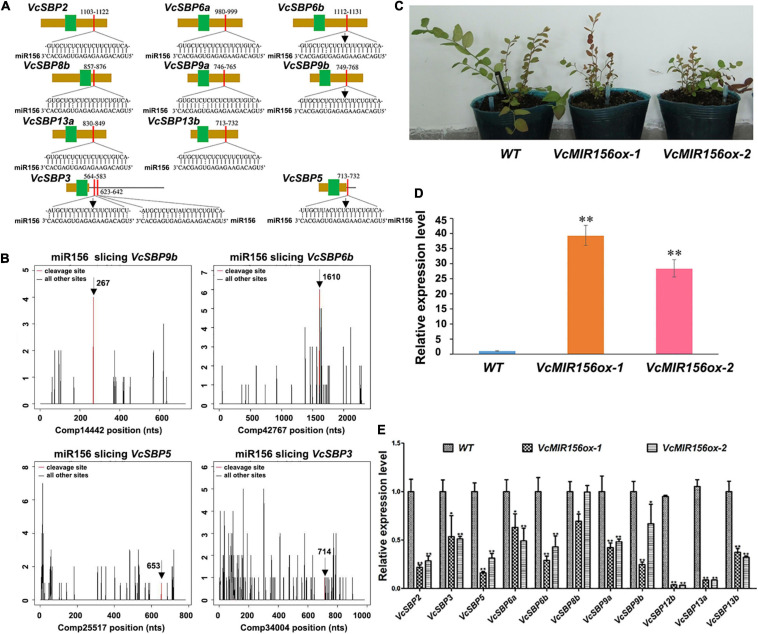
miR156-targeted *SBP* family members in blueberry. **(A)** The diagrams of *VcSBP* sequences targeted by miR156. The brown box, black line, green box, and red box represent CDS sequence, UTR regions, SBP domains, and miR156 responsive elements, respectively. **(B)** Target plots (t-plots) of the miR156-targeted *VcSBP* transcripts (*VcSBP9b*, *VcSBP6b*, *VcSBP5*, and *VcSBP3*). The vertical lines represent the peak of different sliced *VcSBP* transcripts, and the red line denotes the peak of miR156-mediated transcript slices. The exact cleavage sites are indicated by vertical arrows in the **(A,B)**. **(C)** The photograph of transgenic blueberry lines with *VcMIR156a* overexpression and untransformed control (WT). **(D)** Expression analysis of *VcMIR156a* in transgenic blueberry lines. **(E)** The expression pattern of *VcSBP* genes in WT and transgenic blueberry lines overexpressing *VcMIR156a*. Total RNAs extracted from blueberry leaf was used for qRT-PCR analysis. Three biological replicates and three technical replicates for each biological replicate were performed. Error bars indicate standard errors. The data were normalized against the gene *VcACTIN*, and significant differences are denoted by asterisks: ^∗^*P* < 0.05, ^∗∗^*P* < 0.01.

It is well acknowledged that most SBP family members can be regulated through miR156/157-mediated mRNA cleavage or translational repression in plants (Wang and Wang, 2015). Previously we identified six *MIR156/MIR157* genes in blueberry (Hou et al., 2017). To computationally identify the SBP family members targeted by miR156/157 in blueberry, the miRNA responsive elements (MREs) were searched using the complementary sequences of six miR156/157s against the 20 *VcSBP*s and the 2 alternatively spliced variants (Supplementary Figure 3). It was found that 10 of the *VcSBP*s harbor one or two MRE(s) for miR156/157 (Figure 4A), suggesting that they have the potentials to be targeted by miR156/157. Further examination indicated that the MREs were located in the coding region of 8 *VcSBP*s (*VcSBP2*, *VcSBP6a*, *VcSBP6b*, *VcSBP8b*, *VcSBP9a*, *VcSBP9b*, *VcSBP13a*, and *VcSBP13b*) and 3′-UTR region of two *VcSBP*s (*VcSBP3* and *VcSBP5*). Noticeably, two MREs for miR156/157 were observed in the 3′-UTR region of *VcSBP3*. It is worth mentioning that, previously, *VcSBP2/SPL12* was experimentally verified to be targeted by miR156/157 (Li et al., 2020). Here, further mining of our degradome data revealed four additional miR156/157-guided cleavages of *VcSBP* transcripts, including the assembled sequences Comp14442, Comp42467, Comp25517, and Comp34004 (Figure 4B), which correspond to the cDNA sequences of *VcSBP9b*, *VcSBP6b*, *VcSBP5*, and *VcSBP3*, respectively. These data suggest that these *VcSBP*s might be targeted by miR156 *in vivo*.

To verify the SBP family members targeted by miR156/157s *in vivo*, genetic transformation was performed to obtain transgenic blueberry lines overexpressing *VcMIR156a* (Figure 4C). qPCR analysis indicated that the expression of *VcMIR156a* was indeed increased in the two transgenic blueberry lines (Figure 4D). Furthermore, the expressions of the above 10 *SBP*s and *VcSBP12b* were examined in the transgenic blueberry lines and untransformed control. As shown in Figure 4E, all the examined *VcSBP* genes were significantly repressed by the *VcMIR156a* overexpression, especially *VcSBP2*, *VcSBP5*, *VcSBP6b*, *VcSBP9a*, *VcSBP12b*, *VcSBP13a*, and *VcSBP13b* with more than two-fold decreases, suggesting that these eleven *SBP* genes can be regulated through miR156/157-mediated mRNA cleavage *in vivo*.

### *VcSBP* Family Plays Diverse Roles in Arabidopsis and Affects Chlorophyll Accumulation

**FIGURE 5:**
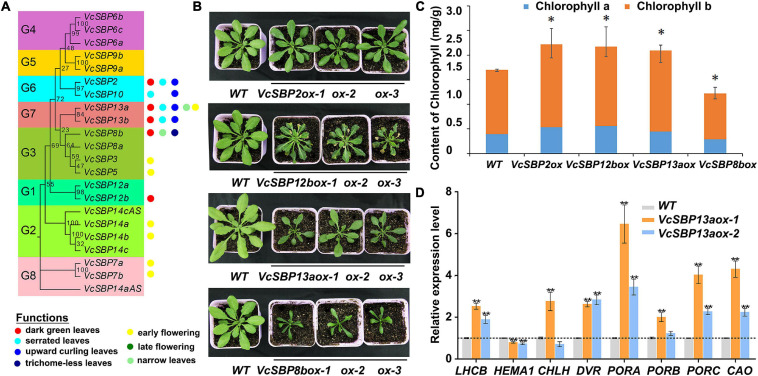
VcSBP family play diverse roles in Arabidopsis and affects chlorophyll accumulation. **(A)** Summery of functional roles of the *VcSBP* family genes in Arabidopsis. **(B)** Photographs of transgenic Arabidopsis lines overexpressing *VcSBP* genes (*VcSBP2*, *VcSBP12b*, *VcSBP13a*, and *VcSBP8b*). **(C)** Chlorophyll contents of wild type and transgenic lines overexpressing *VcSBP2*, *VcSBP12b*, *VcSBP13a*, or *VcSBP8b*. **(D)** Expression patterns of 8 chlorophyll-associated genes (*AtHEMA1*, *AtDVR*, *AtPORA*, *AtPORB*, *AtPORC*, *AtCAO*, *AtCHLH*, and *AtLHCB*) in wild type and transgenic lines overexpressing *VcSBP13a*. Total RNAs were exacted from 14-day-old transgenic seedlings and wild type. Values were normalized against the gene *AtACTIN8*. Error bars in **(C,D)** indicate standard errors of three biological and technical replicates, and significant differences are denoted by asterisks: *P < 0.05, **P < 0.01.

To investigate the functional roles of *VcSBP* genes, transgenic Arabidopsis lines were generated for the *VcSBP* genes. Phenotypic analysis indicated that the *VcSBP* family genes performs diverse functions in Arabidopsis, mainly involved in four aspects of biological or developmental processes: flowering, leaf development, trichome formation, and chlorophyll accumulation. Overexpression of seven *VcSBP*s (*VcSBP7a/7b*, *VcSBP14a/14b*, *VcSBP3*, *VcSBP5*, and *VcSBP13a*) led to early flowering (Figure 5A and Supplementary Figures 4A,B), whereas *VcSBP8b* repressed plant flowering and trichome formation in Arabidopsis (Figure 5A and Supplementary Figures 4C,D). It was also observed that curling leaf could be arisen from overexpression of each of the three *VcSBP* genes, *VcSBP10*, *VcSBP13a*, or *VcSBP13b* (Figure 5A and Supplementary Figure 4E), while the transgenic lines overexpressing *VcSBP13a* or *VcSBP8b* showed narrow leaf (Figures 5A,B). Additionally, serrated leaf was observed in the transgenic lines overexpressing *VcSBP12b* or *VcSBP13a* (Figure 5A and Supplementary Figure 4F). Clearly, the *VcSBP*s in the same phylogenetic clade cannot always generate similar morphological characters (Figure 5A).

Previously, we reported that overexpression of *VcSBP2/SPL12* enhanced chlorophyll accumulation in Arabidopsis (Li et al., 2020). Here, we noticed that transgenic plants overexpressing each of the 4 *VcSBP* genes (*VcSBP2*, *VcSBP12b*, *VcSBP13a*, and *VcSBP8b*) clearly showed dark green leaves (Figure 5B). Also, a little succulence was observed for the leaves of the *VcSBP8b*-overexpressing transgenic lines (Supplementary Figures 4C,D). Chlorophyll contents were then determined in the transgenic Arabidopsis lines. As shown in Figure 5C, total chlorophyll contents in the transgenic lines overexpressing *VcSBP2*, *VcSBP12b*, or *VcSBP13a* were 1.28, 1.31, and 1.24 times higher, respectively, than that in WT. Consistently, both chlorophyll a and b were increased by 1.15–1.43 and 1.24–1.29-folds as compared to WT, respectively (Figure 5C). However, chlorophyll content was decreased in the *VcSBP8b*-overexpressing transgenic lines, which might be due to the succulent leaves. Furthermore, the expressions of eight chlorophyll-associated genes were examined in the *VcSBP13a*-overexpressing transgenic lines, including 7 chlorophyll biosynthetic genes (*AtHEMA1*, *AtDVR*, *AtPORA*, *AtPORB*, *AtPORC*, *AtCAO*, and *AtCHLH*) and one chlorophyll-binding protein gene (*AtLHCB*). Consequently, all the genes were significantly upregulated by overexpression of *VcSBP13a* except *AtHEMA1* that showed a slight decrease (Figure 5D). Especially, *AtPORA* was remarkably increased by 10-folds (Figure 5D). These results indicated that *VcSBP*s affect chlorophyll accumulation via regulating the expression of chlorophyll-associated genes in Arabidopsis. Since *SBP* family is transcriptionally regulated by miR156, the expressions of the above eight chlorophyll biosynthetic genes were examined in the *VcMIR156a*-overexpressing transgenic Arabidopsis. Consistently, *AtDVR* and *AtPORC* were significantly repressed by *VcMIR156a* overexpression (Supplementary Figure 5).

### *SBP* Family Might Affect the Expression of *VcLHCB1* via Targeting Its Promoter in Blueberry

**FIGURE 6:**
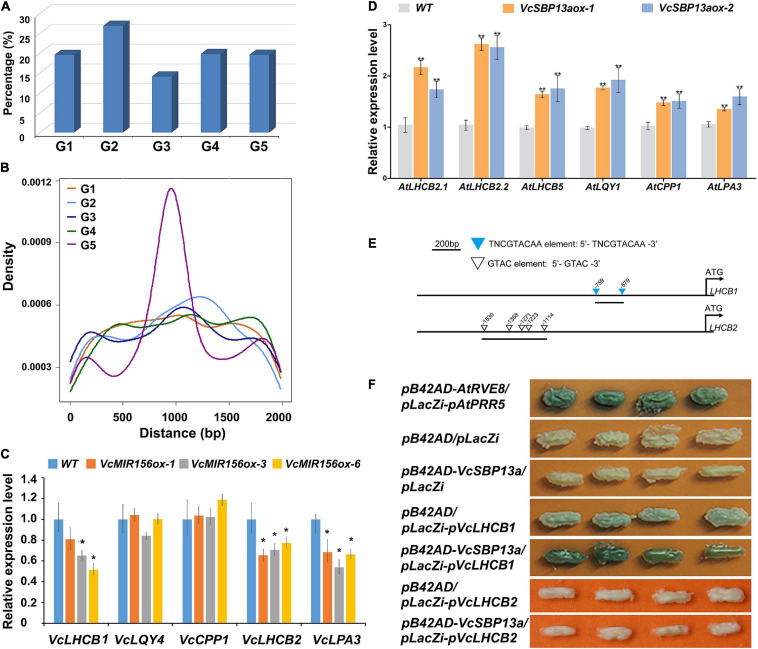
Computational and experimental analysis of the target client(s) of SBP(s) in blueberry. **(A)** The categories of putative target clients. Five categories were classified in terms of their functional roles, including transcription, DNA-or-RNA-related (G1); metabolism defense or protein binding (G2); cellular process (G3); synthesis, catalysis, or modification (G4); and biological process unknown (G5). **(B)** The density of SBP binding sites in the promoter regions of each group of target clients. **(C)** The expression patterns of chlorophyll-associated genes (*VcLHCB1*, *VcLQY4*, *VcCPP1*, *VcLHCB2*, and *VcLPA3*) in WT and transgenic blueberry lines overexpressing *VcMIR156a*. **(D)** The expression patterns of chlorophyll-associated genes (*AtLHCB2.1*, *AtLHCB2.2*, *AtLHCB5*, *AtLQY4*, *AtCPP1*, and *AtLPA3*) in WT and transgenic Arabidopsis lines overexpressing *VcSBP13a*. Values were normalized against the gene *VcACTIN* **(C)** or *AtACTIN8* **(D)**. Error bars indicate standard errors of three biological and technical replicates, and significant differences are denoted by asterisks: *P < 0.05, **P < 0.01. **(E)** The schematic diagrams of the promoters of *VcLHCB1* and *VcLHCB2*. The downward triangles indicate the elements of TNCGTACAA and GTAC, and the number shows the start site of each element. **(F)** Interaction assay between *VcSBP13a* and the promoters of *VcLHCB1* and *VcLHCB2* using the yeast one-hybrid assay. *pB42AD/pLacZi-pVcLHCB1*, *pB42AD/pLacZi-pVcLHCB2*, *pB42AD/pLacZi*, and *pB42AD-VcSBP13a/pLacZi* were set as negative controls; and *pB42AD-AtRVE8/placZi-AtPRR5* as positive control.

Increasing evidence indicated that SBPs are able to bind to the consensus sequence TNCGTACAA with GTAC as its essential core (Kropat et al., 2005). To provide some clues for understanding the targets of SBPs in blueberry, the potential genome-wide binding sites were searched using the consensus sequence against the *V. corymbosum* cv. Draper v1.0 genome. Consequently, 2568 genes were found to harbor the potential binding motif of SBP proteins in their promoter regions (Supplementary Table 4), suggesting that they are possible targets of SBP proteins in blueberry. The potential targets were classified into five groups based on their functional roles, including transcription, DNA-or-RNA-related; metabolism defense or protein binding; cellular process; synthesis, catalysis or modification; biological process unknown (Figure 6A). Further examination indicated that the distribution of the potential binding sites in the promoter regions varied among the five groups. The density of the binding sites over the target genes in the groups G5, G3, and G2 peaked around ∼900, 1100, and 1200 bp upstream of their TSSs, while no obvious peak was found for the target genes in the groups G4 and G1 (Figure 6B).

Consistent with the above results that *VcSBP*s affect chlorophyll accumulation in Arabidopsis, nine chlorophyll-associated genes were found to harbor the potential binding site of SBP proteins, including three *LIGHT HARVESTING CHLOROPHYLL A/B BINDING PROTEIN*s (*VcLHCB*s), two *LOW QUANTUM YIELD OF PHOTOSYSTEM II1* (*VcLQY1*), 3 *CHAPERONE-LIKE PROTEIN OF POR1-like* (*VcCPP1*), and one *LOW PSII ACCUMULATION 3* (*VcLPA3*). Subsequently, five genes were chosen as representatives to examine their expression patterns in transgenic blueberry plants overexpressing *VcMIR156a* where 11 *VcSBP*s were transcriptionally repressed (Figure 4E). As shown in Figure 6C, the expressions of *VcLPA3* and two *VcLHCB*s were significantly downregulated by *MIR156a* overexpression in blueberry, whereas no significant change was observed for *VcLQY1* and *VcCPP1*. Furthermore, the expression patterns of their corresponding Arabidopsis homologs (such as *AtLHCB2.1*, *AtLHCB2.2*, *AtLHCB5*, *AtLQY1*, *AtCPP1*, and *AtLPA3*) were investigated in transgenic Arabidopsis with *VcSBP13a* overexpression. Consequently, all these homologous genes were significantly promoted by *VcSBP13a* overexpression (Figure 6D).

To examine if the SBP proteins bind to these genes, VcSBP13a and two *VcLHCB*s (*VcLHCB1* and *VcLHCB2*) were separately chosen as representatives of baits and preys to perform Y1H analysis. Sequence analysis indicated that the promoter region of *VcLHCB1* contained two typical binding sites (TNCGTACAA element), whereas only GTAC elements were observed in the promoter region of *VcLHCB2* (Figure 6E). As shown in Figure 6F, like the positive control (*pB42AD-AtRVE8/placZi-AtPRR5*), strong blue colonies were observed when VcSBP13a acts as bait and the fragment of *VcLHCB1* promoter as prey. In contrast, very light blue appeared in the colonies containing *pB42AD* as bait and *pLacZi*-*pVcLHCB1* as prey, and no blue color was shown for the other two negative controls (*pB42AD/pLacZi* and *pB42AD-VcSBP13a/pLacZi*). These results indicated that physical interaction occurred between VcSBP13a and the *VcLHCB1* promoter. However, no blue color was observed in the colonies when *pB42AD-VcSBP13a* acted as bait and *pLacZi*-*pVcLHCB2* as prey.

## Discussion

*SBP* genes belong to a small family of plant-specific transcription factors. In the present study, 20 *SBP* genes were identified in blueberry, and the number of VcSBP family members is similar to the ones in Petunia (21), Tartary buckwheat (24), grape (17), and pear (19) (Hou et al., 2013; Li et al., 2013; Zhou et al., 2018; Liu et al., 2019), supporting the notion that the number of *SBP* genes in different plant species is relatively stable during evolution (Liu et al., 2019). Gene family generally arises from gene duplication during evolution, therefore leading to the acquisition of neofunctionalizations and subfunctionalizations as well as the emergence of backup or redundant genes (Preston and Hileman, 2013; Wang and Wang, 2015). Among the 20 identified *VcSBP*s, only two gene pairs (*VcSPB7a* and *VcSBP7b*, *VcSBP14b*, and *VcSBP14c*) might have been derived from segmental duplication, and one pair (*VcSBP8a* and *VcSBP8b*) from tandem amplification (Figure 1C). Noticeably, the *VcSBP* genes in the same group in the phylogenetic tree showed relatively low identity (8–48%) except the two segmental duplication pairs (*VcSPB7a* and *VcSBP7b*, *VcSBP14b*, and *VcSBP14c*, Figures 1C, 2A), suggesting that most *VcSBP*s might be single-copy genes with functional specificity. However, it was estimated that at least three rounds of whole-genome duplication occurred during the evolution of blueberry species (Wang et al., 2020), which are supposed to facilitate the generation of multiple copy genes. It can be explained by at least two reasons: (1) it is still possible that the number of *VcSBP* genes might have been underestimated since the identification of *SBP* genes was conducted on the basis of available transcriptome data; (2) *VcSBP*s might belong to the duplication-resistant genes, which generally return to single-copy status through the duplication-resistant system or genetic drift after suffering duplication events (Wang et al., 2020).

*SBP* family performs diverse functions during plant growth and development. Here, we presented four aspects of evidence to show the functional diversity of the *SBP* family in blueberry. Firstly, it has been proposed that the diversification of gene structures and conservation of motifs may be tightly associated with the functional evolution of *SBP* genes (Salinas et al., 2012; Hou et al., 2013; Li et al., 2013; Li and Lu, 2014; Shalom et al., 2015; Liu et al., 2019). In the present study, it was revealed that the majority of VcSBP family members belonging to the same phylogenetic group showed similar motif compositions and gene structures, while the diversity in motif compositions and gene structures was observed between the SBP family members in different phylogenetic groups (Figure 2). These observations support a scenario that the SBP family underwent functional conservation and diversification during evolution (Preston and Hileman, 2013; Wang and Wang, 2015; Zhang et al., 2015). Secondly, the spatio-temporal expression is generally thought as key contributors to functional specificity for a gene family (Paul et al., 2012; Hasan et al., 2017). *VcSBP* genes displayed tissue-specific and fruit development stage-specific expression patterns (Figure 3), implying that the *VcSBP* family might exert diverse functions in blueberry. Thirdly, the SBP family can be regulated via miR156-guided transcript cleavage or translational repression, and a subset of *SBP*s have been proved to be targeted by miR156 through recognizing MREs on their transcripts, for example, 11 out of the 17 *SBP*s in Arabidopsis, 15 out of the 27 *SBP*s in apple and 7 out of the 19 *SBP*s in pear (Li et al., 2013; Zhang et al., 2015; Qian et al., 2017). In the present study, several members of the *VcSBP* family were computationally and experimentally demonstrated to be targets of miR156 (Figure 4), implying that a subset of *VcSBP*s are able to form a regulatory hub with miR156, therefore exerting vital functions during blueberry growth and development. Lastly, overexpression of *VcSBP*s in Arabidopsis gave rise to multiple morphological phenotypes (Figure 5 and Supplementary Figure 4). Thus, our results provided an overall framework for understanding the functional diversity of *VcSBP* genes, which will contribute to the genetic improvement of the agronomic traits of blueberry.

The SBP family acts as pivotal regulators of diverse biological and physiological processes in plants. In the present study, functional analysis in Arabidopsis indicated that *VcSBP* genes might be involved in multiple developmental processes such as leaf shape regulation (serrated leaf formation, *VcSBP12b/13a*; narrow leaf, *VcSBP8b/13a*), trichome formation (*VcSBP8b*), and flowering time control (*VcSBP7a/7b/14a/14b/3/5/13a*, Figure 5 and Supplementary Figure 4). These observations are consistent with previous reports in Arabidopsis. For example, *AtSPL10* overexpression causes narrow leaf in Arabidopsis (Gao et al., 2018); *AtSPL3/4/5* exert important functions in regulating Arabidopsis flowering and developmental transition (Jung et al., 2016; Xu et al., 2016); *AtSPL3/4/5/8/9/10/13* affect trichome formation (Yu et al., 2010); and loss-of-function mutation of *AtSPL14* increases the number of leaf hydathodes and enhances leaf margin serration (Stone et al., 2005). Thus, our results support the notion that the functionality of the SBP family proteins is highly conserved among distinct plant species (Preston and Hileman, 2013). However, not all the *VcSBP*s display the same functional roles as their counterparts in other plant species. For instance, overexpression of *VcSBP8b* leads to a very narrow leaf in Arabidopsis (Figure 5B), whereas its Arabidopsis counterpart *AtSPL8* fails to generate similar leaf morphology, and it is *AtSPL10* instead that was reported to modulate leaf morphology (Gao et al., 2018). Previous studies also indicated that mutation of *LG1*, the closest homolog of Arabidopsis *AtSPL8*, in maize, rice, and barley gave rise to the lack of ligules and auricles (Lee et al., 2007; Wang and Wang, 2015), whereas in Arabidopsis mutation of *AtSPL8* fails to cause a similar structure of ligules. Thus, it seems that it is not always possible to foretell the functional roles of individual *SBP* genes based on homology, although the *SBP* family as a whole shows functional conservation across diverse plant species. More interestingly, overexpression of three *VcSBP* genes (*VcSBP10/13a/13b*) in Arabidopsis causes the formation of curling leaves (Supplementary Figure 4E). Previous report indicated that mutation in *rSPL13* led to an up-curled leaf phenotype in alfalfa (Gao et al., 2018). Nevertheless, no evidence shows the formation of curling leaves by being members of the SBP family in Arabidopsis. Thus, it appears that *SBP* family might show species-dependent functions or novel function in some specific plant species.

Several studies indicated that the SBP family plays important roles during fruit ripening. For example, *VmTDR4* (a SQUAMOSA-class *MADS-box* gene) is positively involved in the regulation of anthocyanin accumulation during bilberry fruit ripening (Jaakola et al., 2010), while *MaSPL16* in banana regulates carotenoid biosynthesis through promoting the expression of *MaLCYB*s (Zhu et al., 2020). Likewise, *SlSPL-CNR*, an SBP transcription factor in tomato, is mainly expressed in ripening fruits and serves as a positive player in the regulation of fruit ripening and cell death (Lai et al., 2020). In the present study, five *VcSBP* genes were found to be expressed at relatively low levels at three early stages of fruit development and significantly increased during fruit ripening (Figure 3I), suggesting that they might act as regulatory hubs to control fruit ripening in blueberry. In contrast, 10 *VcSBP* genes were highly expressed at three early stages of fruit development and dramatically decreased to a low level when fruit initiates ripening (Figure 3G,H), which is consistent with previous reports that the expressions of *VvSPL6/10/13* were gradually decreased as grape berry develops and ripens (Cui et al., 2018), while the *FvSPL*s were transcriptionally decreased during strawberry fruit ripening (Xiong et al., 2018). These results suggest that the 10 *VcSBP*s might be required for fruit development and suppressed during fruit ripening in blueberry.

Generally, the development and ripening of fleshy fruits are accompanied by a wide range of changes at cellular, molecular and metabolic levels, including fruit enlargement, degreening, accumulation of pigments, softening, etc. Previously, we revealed that *VcSBP2/SPL12* affects the accumulation of chlorophylls in Arabidopsis (Li et al., 2020). In the present study, the contents of chlorophyll a and b were found to be increased by the overexpression of at least three *SBP* genes (*VcSBP2*, *VcSBP12a*, and *VcSBP13a*) in Arabidopsis (Figure 5). Moreover, the chlorophyll biosynthetic genes in Arabidopsis were indeed elevated by *VcSBP13a* overexpression (Figure 5D). These observations indicated that a subset of *VcSBP*s might be involved in the regulation of chlorophyll accumulation. Previous studies have revealed that SBP family proteins can directly interact with their clients (for example, *AtFUL*, *AtSOC1*, *AtDFR*, *AtAP1*, *MdWRKY100*, *MaLCYB1.1*, and *MaLCYB1.2*, *MADS5*, and *MADS32*), thereby regulating diverse biological processes in plants such as flowering, inflorescence formation, biosynthesis of secondary metabolites, root regeneration, and response to stress (Yamaguchi et al., 2009; Gou et al., 2019; Ma et al., 2020; Zhu et al., 2020). However, the targets of SBP proteins associated with chlorophyll accumulation have remained to be found. Our Y1H assay showed that VcSBP13a could physically bind to the promoter region of an *LHCB* gene in blueberry (Figure 6). Thus, we proposed that VcSBPs are able to positively regulate the expressions of chlorophyll-associated genes (at least *VcLHCB1*) directly through binding to their promoter regions to affect chlorophyll accumulation in blueberry.

In conclusion, the SBP family was systematically identified and functionally characterized in blueberry, and they show conservation and divergence in characteristics and functional roles across plant species. Based on the targets and functional roles of *VcSBP*s as well as their expression patterns, we propose that a subset of *VcSBP*s might be involved in the regulation of chlorophyll accumulation directly through targeting to the chlorophyll-associated genes such as *VcLHCB1*. These findings provide the first comprehensive understandings of the features and functional diversity of the SBP family in blueberry, which will facilitate their utilization in the improvement of the agronomic traits of blueberry.

## Data Availability Statement

The datasets presented in this study can be found in online repositories. The names of the repository/repositories and accession number(s) can be found in the article/Supplementary Material.

## Author Contributions

SB and XL designed the experiments. XX, SY, BS, HL, and PY performed the experiments. JW and SL performed the data analyzes. SB, XL, and YC wrote the manuscript. All authors read and approved the final manuscript.

## Conflict of Interest

The authors declare that the research was conducted in the absence of any commercial or financial relationships that could be construed as a potential conflict of interest.

## Footnotes

1 https://www.arabidopsis.org/

2 https://phytozome.jgi.doe.gov/pz/portal.html

3 www.vaccinium.org

4 https://prosite.expasy.org/

5 https://web.expasy.org/compute_pi/

6 http://weblogo.threeplusone.com/

7 https://gsds.cbi.pku.edu.cn/

8 http://meme-suite.org/tools/meme

9 http://plantgrn.noble.org/psRNATarget/

10 http://eggnog-mapper.embl.de/

11 www.ncbi.nlm.nih.gov/Structure/cdd/wrpsb.cgi

12 https://www.vaccinium.org/analysis/49

## Supplementary Material

Supplementary Figure 1 Phylogenetic analysis of *SBP* genes in blueberry and Arabidopsis. The CDS sequences of *AtSBP*s were downloaded from the TAIR website (www.arabidopsis.org). A neighbor-joining tree was generated with the MEGA7 software using the CDS sequences of the *SBP* genes in blueberry and Arabidopsis.

Supplementary Figure 2 Phylogenetic relationship of VcSBP proteins with the SBPs in other plant species. A maximum likelihood tree was generated with the MEGA X software using the putative amino acid sequences of 101 SBP proteins, which were clustered into eight groups (G1-G6). The SBP proteins in the same species are represented with the same symbol: blue check, *V. corymbosum*; orange circle, *Vitis vinifera*; yellow square, *Malus domestica*; pink star, *Solanum lycopersicum*; gray triangle, *Arabidopsis thaliana*.

Supplementary Figure 3 Alignment of miR156 complementary sequences within *VcSBP* genes. **(A)** Complementary sequences are within coding regions and the 3′UTR. Reverse complement sequences of the mature vco-miR156a, vco-miR156b-5p, vco-miR156c-5p, vco-miR156e-5p, vco-miR156g-5p, and vco-miR157a genes are shown below the alignment for comparison. **(B)** The sequence logo of miR156 responsive elements in *VcSBP* genes. The total height of each stack represents the conservation degree of each position, while the height of the letters within each stack indicates the relative frequency of the corresponding amino acid.

Supplementary Figure 4 Phenotype of the transgenic Arabidopsis individually overexpressing the *VcSBP* family genes. Early **(A, B)** – and – late **(C)** flowering phenotypes of the transgenic Arabidopsis overexpressing the gene *VcSBP7a*, *VcSBP7b*, *VcSBP14a*, *VcSBP14b*, *VcSBP3*, *VcSBP5*, *VcSBP13a*, or *VcSBP8b*. **(D)** Less trichome phenotype of the transgenic Arabidopsis overexpressing the gene *VcSBP7a*. **(E)** Curled leaf phenotype of the transgenic Arabidopsis overexpressing the gene *VcSBP10*, *VcSBP13a*, or *VcSBP13b*. **(F)** Serrated leaf phenotype of the transgenic Arabidopsis overexpressing the gene *VcSBP13a* or *VcSBP12b*.

Supplementary Figure 5 Expression patterns of 8 chlorophyll-associated genes (*AtLHCB*, *AtHEMA1*, *AtCHLH*, *AtDVR*, *AtPORA*, *AtPORB*, *AtPORC*, and *AtCAO*) in wild type and transgenic lines overexpressing *VcMIR156a*. Total RNAs were exacted from 7-day-old transgenic seedlings and wild type. Values were normalized against the gene *AtACTIN8*. Error bars indicate standard errors of three biological and technical replicates, and significant differences are denoted by asterisks: **P* < 0.05, ***P* < 0.01.

Supplementary Table 1 Primers used in the study.

Supplementary Table 2 Characterization of the SBP family in blueberry.

Supplementary Table 3 Similarity and identity between different VcSBP family members.

Supplementary Table 4 The predicted targets of the VcSBP family proteins in blueberry.
