# Supplementary material for: Comprehensive Analysis of the SBP Family in Blueberry and Their Regulatory Mechanism Controlling Chlorophyll Accumulation
Source: Front Plant Sci. 2021 Jul 1;12:703994. doi: 10.3389/fpls.2021.703994 (PMC8281205; doi:10.3389/fpls.2021.703994)
Supplement: Supplementary Figure 1 — Phylogenetic analysis of SBP genes in blueberry and Arabidopsis. The CDS sequences of AtSBPs were downloaded from the TAIR website (www.arabidopsis.org). A neighbor-joining tree was generated with the MEGA7 software using the CDS sequences of the SBP genes in blueberry and Arabidopsis. [file Image_1.pdf]

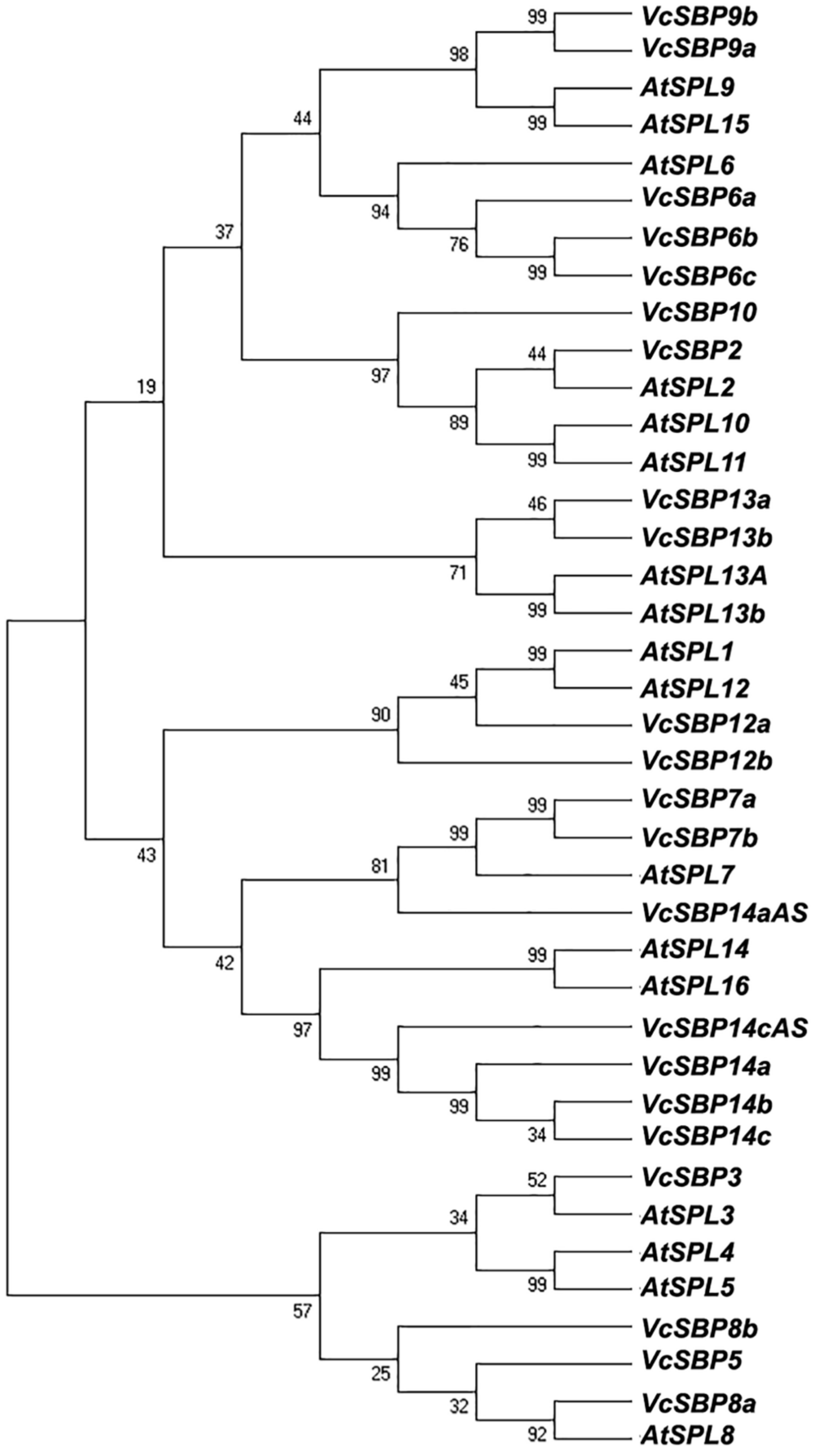
VcSBP9b
VcSBP9a
AtSPL9
AtSPL15
AtSPL6
VcSBP6a
VcSBP6b
VcSBP6c
VcSBP10
VcSBP2
AtSPL2
AtSPL10
AtSPL11
VcSBP13a
VcSBP13b
AtSPL13A
AtSPL13b
AtSPL1
AtSPL12
VcSBP12a
VcSBP12b
VcSBP7a
VcSBP7b
AtSPL7
VcSBP14aAS
AtSPL14
AtSPL16
VcSBP14cAS
VcSBP14a
VcSBP14b
VcSBP14c
VcSBP3
AtSPL3
AtSPL4
AtSPL5
VcSBP8b
VcSBP5
VcSBP8a
AtSPL8
99
98
99
44
94
76
99
37
44
97
89
99
19
46
71
99
99
45
90
99
99
81
43
42
99
97
99
99
34
52
34
99
57
25
32
92

Supplementary Figure 1 Phylogenetic analysis of *SBP* genes in blueberry and Arabidopsis. The CDS sequences of *AtSBP*s were downloaded from the TAIR website (www.arabidopsis.org). A neighbor-joining tree was generated with the MEGA7 software using the CDS sequences of the *SBP* genes in blueberry and Arabidopsis.
