# Supplementary material for: Comprehensive Analysis of the SBP Family in Blueberry and Their Regulatory Mechanism Controlling Chlorophyll Accumulation
Source: Front Plant Sci. 2021 Jul 1;12:703994. doi: 10.3389/fpls.2021.703994 (PMC8281205; doi:10.3389/fpls.2021.703994)
Supplement: Supplementary Figure 2 — Phylogenetic relationship of VcSBP proteins with the SBPs in other plant species. A maximum likelihood tree was generated with the MEGA X software using the putative amino acid sequences of 101 SBP proteins, which were clustered into eight groups (G1-G6). The SBP proteins in the same species are represented with the same symbol: blue check, V. corymbosum; orange circle, Vitis vinifera; yellow square, Malus domestica; pink star, Solanum lycopersicum; gray triangle, Arabidopsis thaliana. [file Image_2.pdf]

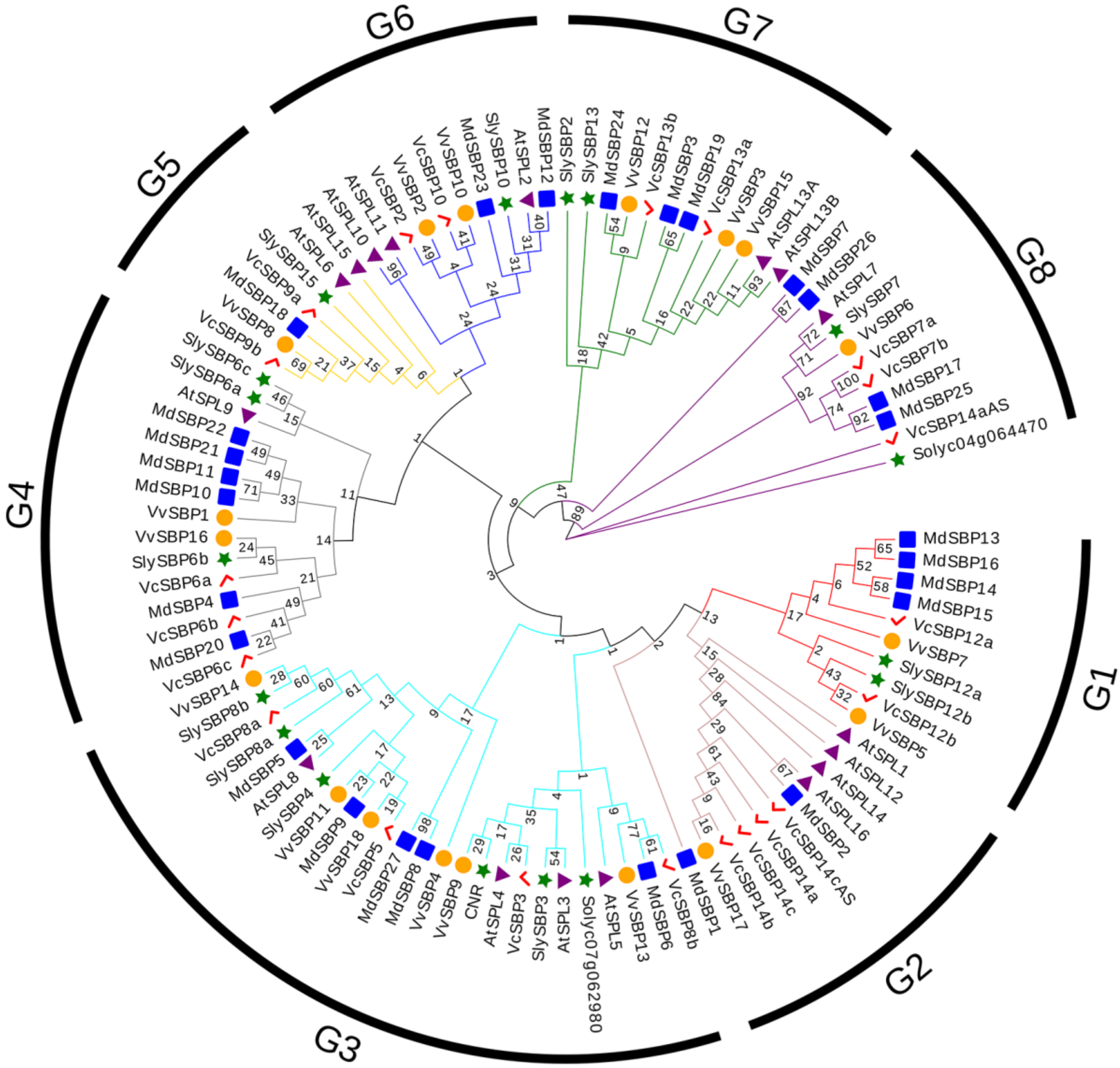

Supplementary Figure 2 Phylogenetic relationship of VcSBP proteins with the SBPs in other plant species. A maximum likelihood tree was generated with the MEGA X software using the putative amino acid sequences of 101 SBP proteins, which were clustered into eight groups (G1-G6). The SBP proteins in the same species are represented with the same symbol: blue check, *Vaccinium corymbosum*; orange circle, *Vitis vinifera*; yellow square, *Malus domestica*; pink star, *Solanum lycopersicum*; grey triangle, *Arabidopsis thaliana*.
