# Supplementary material for: Comprehensive Analysis of the SBP Family in Blueberry and Their Regulatory Mechanism Controlling Chlorophyll Accumulation
Source: Front Plant Sci. 2021 Jul 1;12:703994. doi: 10.3389/fpls.2021.703994 (PMC8281205; doi:10.3389/fpls.2021.703994)
Supplement: Supplementary Figure 3 — Alignment of miR156 complementary sequences within VcSBP genes. (A) Complementary sequences are within coding regions and the 3′UTR. Reverse complement sequences of the mature vco-miR156a, vco-miR156b-5p, vco-miR156c-5p, vco-miR156e-5p, vco-miR156g-5p, and vco-miR157a genes are shown below the alignment for comparison. (B) The sequence logo of miR156 responsive elements in VcSBP genes. The total height of each stack represents the conservation degree of each position, while the height of the letters within each stack indicates the relative frequency of the corresponding amino acid. [file Image_3.pdf]

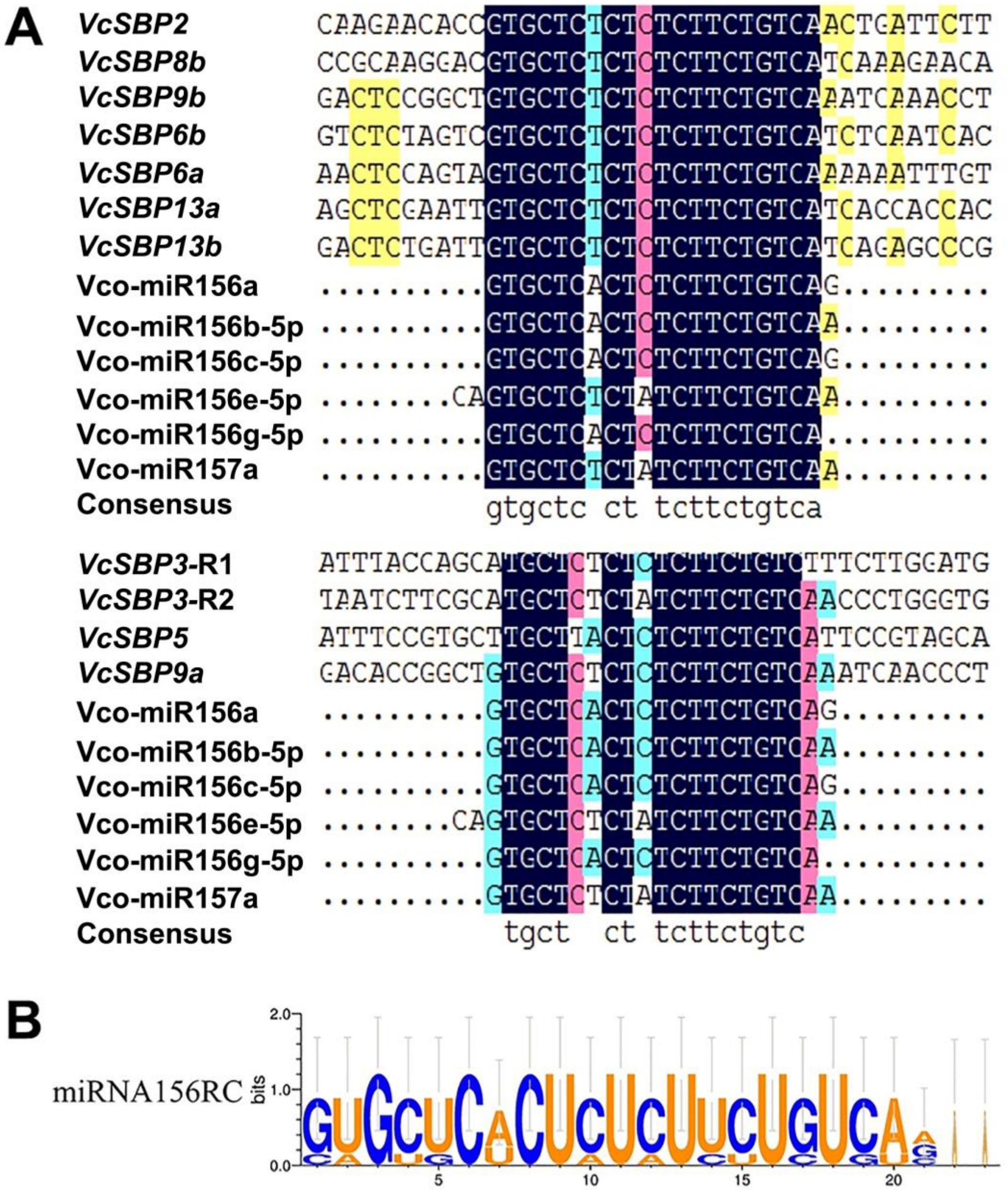

Supplementary Figure 3 Alignment of miR156 complementary sequences within *VcSBP* genes. **(A)** Complementary sequences are within coding regions and the 3' UTR. Reverse complement sequences of the mature vco-miR156a, vco-miR156b-5p, vco-miR156c-5p, vco-miR156e-5p, vco-miR156g-5p, and vco-miR157a genes are shown below the alignment for comparison. **(B)** The sequence logo of miR156 responsive elements in *VcSBP* genes. The total height of each stack represents the conservation degree of each position, while the height of the letters within each stack indicates the relative frequency of the corresponding nucleotide.
