# Supplementary material for: Comprehensive Analysis of the SBP Family in Blueberry and Their Regulatory Mechanism Controlling Chlorophyll Accumulation
Source: Front Plant Sci. 2021 Jul 1;12:703994. doi: 10.3389/fpls.2021.703994 (PMC8281205; doi:10.3389/fpls.2021.703994)
Supplement: Supplementary Figure 4 — Phenotype of the transgenic Arabidopsis individually overexpressing the VcSBP family genes. Early (A, B) – and – late (C) flowering phenotypes of the transgenic Arabidopsis overexpressing the gene VcSBP7a, VcSBP7b, VcSBP14a, VcSBP14b, VcSBP3, VcSBP5, VcSBP13a, or VcSBP8b. (D) Less trichome phenotype of the transgenic Arabidopsis overexpressing the gene VcSBP7a. (E) Curled leaf phenotype of the transgenic Arabidopsis overexpressing the gene VcSBP10, VcSBP13a, or VcSBP13b. (F) Serrated leaf phenotype of the transgenic Arabidopsis overexpressing the gene VcSBP13a or VcSBP12b. [file Image_4.pdf]

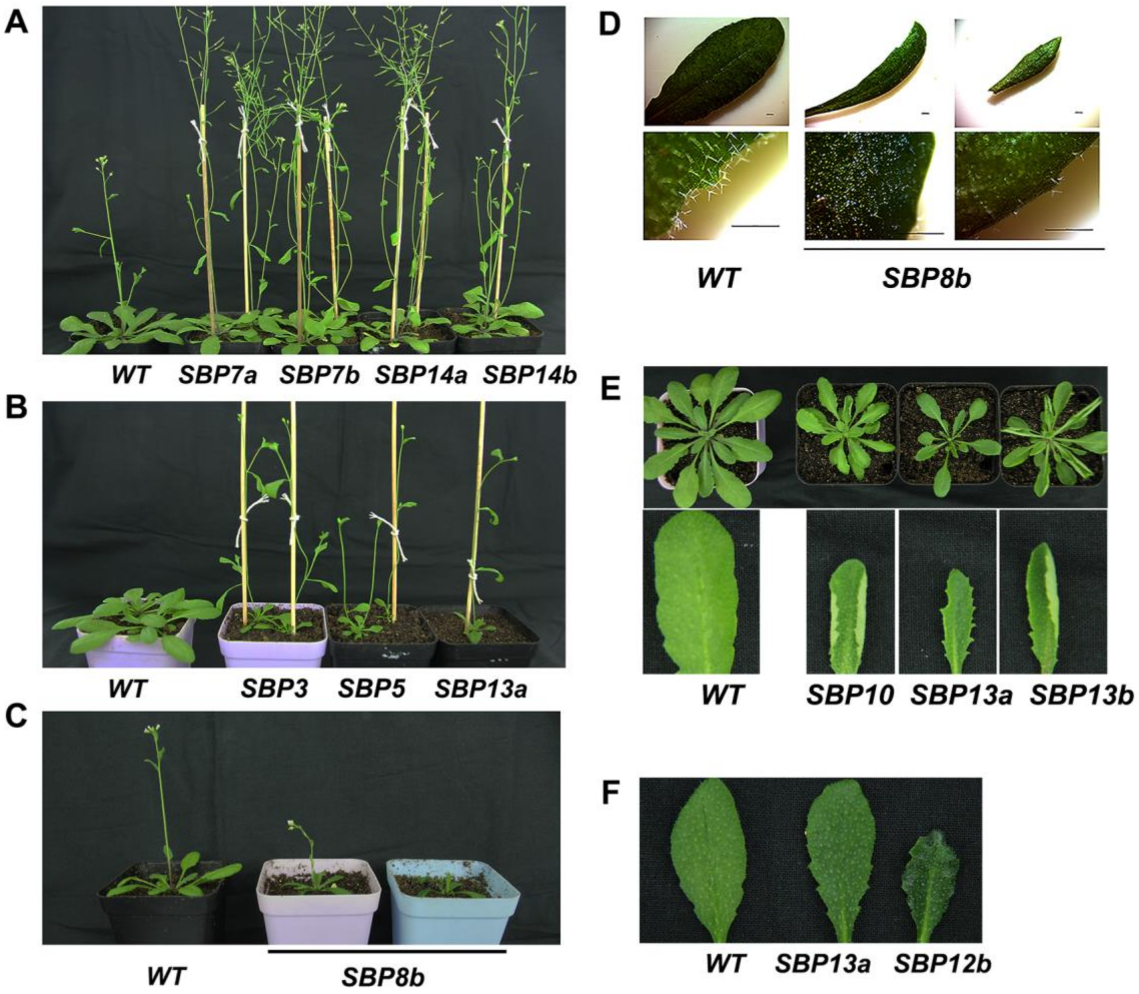

Supplementary Figure 4 Phenotype of the transgenic Arabidopsis individually overexpressing the *VcSBP* family genes. Early **(A-B)** - and - late **(C)** flowering phenotypes of the transgenic Arabidopsis overexpressing the gene *VcSBP7a*, *VcSBP7b*, *VcSBP14a*, *VcSBP14b*, *VcSBP3*, *VcSBP5*, *VcSBP13a* or *VcSBP8b*. **(D)** Less trichome phenotype of the transgenic Arabidopsis overexpressing the gene *VcSBP7a*. **(E)** Curled leaf phenotype of the transgenic Arabidopsis overexpressing the gene *VcSBP10*, *VcSBP13a* or *VcSBP13b*. **(F)** Serrated leaf phenotype of the transgenic Arabidopsis overexpressing the gene *VcSBP13a* or *VcSBP12b*.
