# Supplementary material for: Comprehensive Analysis of the SBP Family in Blueberry and Their Regulatory Mechanism Controlling Chlorophyll Accumulation
Source: Front Plant Sci. 2021 Jul 1;12:703994. doi: 10.3389/fpls.2021.703994 (PMC8281205; doi:10.3389/fpls.2021.703994)
Supplement: Supplementary Figure 5 — Expression patterns of 8 chlorophyll-associated genes (AtLHCB, AtHEMA1, AtCHLH, AtDVR, AtPORA, AtPORB, AtPORC, and AtCAO) in wild type and transgenic lines overexpressing VcMIR156a. Total RNAs were exacted from 7-day-old transgenic seedlings and wild type. Values were normalized against the gene AtACTIN8. Error bars indicate standard errors of three biological and technical replicates, and significant differences are denoted by asterisks: *P < 0.05, **P < 0.01. [file Image_5.pdf]

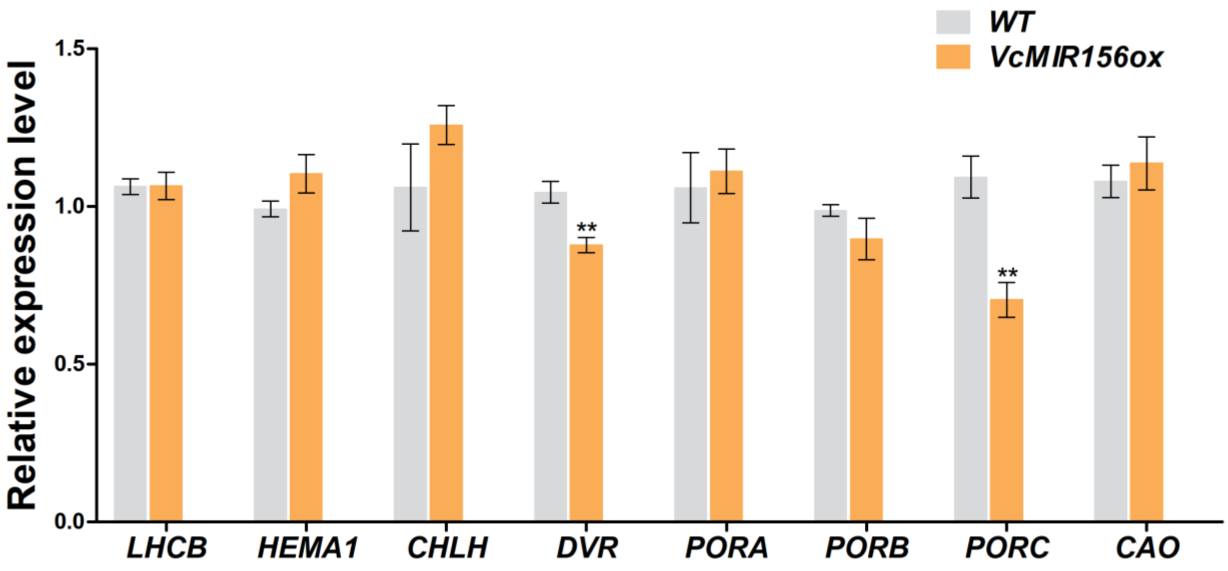

Supplementary Figure 5 Expression patterns of 8 chlorophyll-associated genes (*AtLHCB*, *AtHEMA1*, *AtCHLH*, *AtDVR*, *AtPORA*, *AtPORB*, *AtPORC*, *AtCAO*) in wild type and transgenic lines overexpressing *VcMIR156a*. Total RNAs were exacted from 7-day-old transgenic seedlings and wild type. Values were normalized against the gene *AtACTIN8*. Error bars indicate standard errors of three biological and technical replicates, and significant differences are denoted by asterisks: *P<0.05, **P<0.01.
