# Supplementary material for: Comprehensive Analysis of the SBP Family in Blueberry and Their Regulatory Mechanism Controlling Chlorophyll Accumulation
Source: Front Plant Sci. 2021 Jul 1;12:703994. doi: 10.3389/fpls.2021.703994 (PMC8281205; doi:10.3389/fpls.2021.703994)
Supplement: Supplementary Table 2 — Characterization of the SBP family in blueberry. [file Table_2.DOC]

**Table S2** Characterization of the *SBP* gene family in blueberry.

| Name | Scaffold | Location | CDS length (bp) | Protein | | | Introns |
| --- | --- | --- | --- | --- | --- | --- | --- |
|  |  |  |  | Size (aa) | MW (kDa) | pI |  |
| VcSBP2 | 2 | VaccDscaff2: 24,157,421-24,162,501 (+) | 1380 | 459 | 50.74 | 8.82 | 3 |
| VcSBP3 | 24 | VaccDscaff24: 23,976,532-23,978,475 (+) | 465 | 154 | 17.03 | 6.60 | 1 |
| VcSBP5 | 883 | VaccDscaff883: 12,450-15,376 (-) | 564 | 187 | 20.58 | 9.05 | 1 |
| VcSBP6a | 25 | VaccDscaff25: 28,525,950-28,528,588 (+) | 1338 | 445 | 49.48 | 9.12 | 2 |
| VcSBP6b | 4 | VaccDscaff4: 34,772,761-34,779,957 (+) | 1545 | 514 | 56.74 | 6.76 | 3 |
| VcSBP6c | 35 | VaccDscaff35: 25,525,425-25,526,060 (+) | 636 | 211 | 23.17 | 8.74 | 0 |
| VcSBP7a | 8 | VaccDscaff8: 37,121,061-37,128,989 (-) | 2469 | 822 | 92.64 | 5.99 | 9 |
| VcSBP7b | 5 | VaccDscaff5: 4,008,574-4,016,625 (-) | 2457 | 818 | 92.16 | 5.96 | 9 |
| VcSBP8a | 22 | VaccDscaff22: 4,672,162-4,674,174 (-) | 990 | 329 | 36.04 | 8.83 | 2 |
| VcSBP8b | 22 | VaccDscaff22: 4,652,428-4,657,052 (+) | 1227 | 408 | 44.65 | 7.74 | 2 |
| VcSBP9a | 5 | VaccDscaff5: 28,033,197-28,037,175 (-) | 1077 | 358 | 39.10 | 9.39 | 2 |
| VcSBP9b | 26 | VaccDscaff26: 35,087,168-35,093,813 (-) | 1098 | 365 | 38.76 | 8.99 | 2 |
| VcSBP10 | 23 | VaccDscaff23: 6,685,852-6,687,086 (+) | 765 | 254 | 28.50 | 8.88 | 1 |
| VcSBP12a | 11 | VaccDscaff11: 39,841,489-39,849,774 (-) | 3036 | 1011 | 111.91 | 6.00 | 9 |
| VcSBP12b | 1 | VaccDscaff1: 307,303 -310,655 (+) | 1083 | 360 | 39.35 | 7.59 | 3 |
| VcSBP13a | 34 | VaccDscaff34: 7,256,375-7,259,198 (+) | 1104 | 367 | 40.30 | 8.63 | 2 |
| VcSBP13b | 4 | VaccDscaff4: 26,156,630-26,158,444 (+) | 969 | 322 | 35.23 | 8.26 | 2 |
| VcSBP14a | 23 | VaccDscaff23: 36,665,010-36,669,614 (+) | 3222 | 1073 | 117.97 | 6.66 | 9 |
| VcSBP14aAS | 23 | VaccDscaff23: 36,665,713-36,666,075 (+) | 363 | 120 | 13.65 | 10.54 | 0 |
| VcSBP14b | 12 | VaccDscaff12: 1,852,439-1,854,507 (+) | 1713 | 570 | 61.84 | 6.19 | 2 |
| VcSBP14c | 12 | VaccDscaff12: 2,851,560-2,853,775 (-) | 1755 | 584 | 63.62 | 6.35 | 3 |
| VcSBP14cAS | 12 | VaccDscaff12: 2,853,134-2,853,775 (-) | 642 | 213 | 23.79 | 8.12 | 0 |
